# Sex differences in patients’ referral to a headache unit

**DOI:** 10.3389/fneur.2025.1755865

**Published:** 2026-01-14

**Authors:** Elena Varas-Martín, Isabel Ros-González, Álvaro Sierra-Mencía, Andrea Recio-García, Paula Caballero-Lillo, Yésica González-Osorio, Rocío Areses-Calderón, Carmen Sánchez-Rodríguez, M. Luz. Peñas-Martínez, Alicia Gonzalez-Martinez, Ángel L. Guerrero-Peral

**Affiliations:** 1 Headache Unit, Neurology Department, Hospital Clínico Universitario de Valladolid, Valladolid, Spain; 2 Hospital Universitario de la Princesa, Madrid, Spain; 3 Instituto de Investigación Sanitaria Princesa (IIS-Princesa), Madrid, Spain; 4 Universidad Autónoma de Madrid (UAM), Madrid, Spain; 5 Department of Medicine, Universidad de Valladolid, Valladolid, Spain; 6 Instituto de Investigación Sanitaria de Valladolid (IBioVALL), Valladolid, Spain

**Keywords:** gender bias, health care disparities, migraine, referral patterns, sex factors

## Abstract

**Background:**

Migraine is two to three times more prevalent in women than in men, yet sex-related differences in referral patterns and treatment pathways remain insufficiently studied. The objective of the study was to analyze sex distribution upon arrival at a Headache Unit. Secondary objectives included evaluation of age differences, time to first consultation and treatment management.

**Methods:**

We conducted a retrospective observational study based on a prospectively collected registry of patients diagnosed with migraine at the Headache Unit of a tertiary hospital, between January 2008 and January 2025. Variables included demographic data such as sex or age at referral, headache variables including years since migraine onset and previous use of symptomatic or preventive treatment as well as primary care or secondary care referral.

**Results:**

Among 6,220 patients with migraine 4,956 (79.7%) were female. Female patients were referred at an older age (39.9 vs. 37.1 years; *p* = 0.004) with longer disease duration (18.1 vs. 14.9 years; *p* < 0.0001). They were more likely to have received triptans (28.6% vs. 21.5%) and preventive treatment (39.5% vs. 31.8%) prior to referral (*p* < 0.001 for all). These trends persisted among patients referred from primary care.

**Conclusion:**

Female patients were referred to a Headache Unit at an older age and after a longer disease duration, while men were less likely to have received specific migraine treatments such as triptans or preventives. These findings highlight relevant sex-based differences in referral patterns and treatment exposure among patients with migraine.

## Background

Scientific evidence has historically been shaped by studies conducted predominantly in male populations, with women—especially of childbearing age—often excluded from clinical trials for safety reasons. This practice centred medical knowledge on male physiology and laid the foundation of evidence-based medicine (1). Although inclusion initiatives began in the 1990s, sex-stratified analyses were rarely performed, and conclusions remained unchanged (2).

Over time, sex and gender differences have become increasingly recognized as essential factors in understanding the epidemiology, pathophysiology, and clinical expression of diseases. Sex refers to biological characteristics (chromosomes, gonads, genitals) (3), while gender reflects sociocultural traits that distinguish masculine from feminine, including roles, norms, and relationships.

This distinction has revealed important disparities across multiple conditions. Autoimmune diseases such as systemic lupus erythematosus predominantly affect female patients (4); cardiovascular disease, while more common in male patients at younger ages, often presents later and atypically in women, contributing to underdiagnosis and worse outcomes (5). Likewise, depression and anxiety are more prevalent in female patients (6), whereas substance use disorders are more common in men but may cause more severe health consequences in women (7). These findings highlight the importance of integrating sex- and gender-based perspectives in research and clinical care (1).

Within the field of migraine, sex and gender considerations are especially relevant. Migraine is two to three times more common in women than in men (8). Prevalence is similar in childhood, but after puberty it rises sharply in female patients, peaking around age 30 (9, 10), with a second peak near age 50 before declining.

Differences also exist in the peak incidence of the disease. Migraine typically reaches its highest incidence later in women (20–24 years) than in men (15–19 years) (11), and men tend to experience longer remission periods (12).

The clinical course further illustrates sex differences. Evidence is mixed regarding whether female sex increases the risk of progression to chronic migraine (13), but women are more likely to experience more frequent, longer, and more severe attacks, with greater disability (14). They also more commonly report associated symptoms such as nausea, vomiting, phonophobia, photophobia, and cutaneous allodynia (8). Gender norms may additionally bias reporting, as men could be less likely to acknowledge extreme pain (15, 16).

Treatment guidelines, however, generally do not differentiate by sex. Current recommendations for acute and preventive management are the same, except for precautions during pregnancy (17) and potential interactions with contraceptives. Despite this, women are more likely than men to receive acute and preventive therapies (18). Nevertheless, sex- and gender-based medicine still lacks a central role in clinical practice.

We therefore hypothesized that sex associated traditional gender roles may influence migraine diagnosis and treatment in our region. The main objective was to analyse sex distribution upon arrival at a Headache Unit, with secondary objectives including differences in age, time to first consultation, and treatment management.

## Materials and methods

We performed an observational study with a retrospective analysis conducted on a prospectively collected registry of patients with migraine firstly attended in a Headache Unit of University Clinical Hospital in Valladolid, Spain, between January 2008 and January 2025.

Patients were referred mainly by general practitioners, and also by general neurologists and other medical specialists from the hospital. The recommendations for referral from Primary Care were patients who did not respond to symptomatic treatment, or those in whom at least two medications had been tried as preventive treatment. On the other hand, general neurologists and other specialists could refer patients to the Headache Unit when deemed necessary. All patients were diagnosed by a neurologist with experience in Headache Disorders accordingly to the International Classification of Headache Disorders (ICHD), using the current version at the time of assessment (ICHD-3) (19), although the previous version (ICHD-2) (20) was applied during the earlier years of the study. Patients with other primary headache disorders or with secondary headaches were excluded. Missing data were under 20% for the main variables included in the analyses.

For the stratified analysis, patients were categorized into four age groups: 0–12, 12–30, 30–50, and >50 years. The cut-off of 12 years was chosen because it corresponds to the transition from childhood to adolescence, a stage at which migraine patterns, healthcare-seeking behavior, and treatment approaches can substantially differ (21). The additional categories (12–30, 30–50, >50) were defined to reflect clinically meaningful life stages with potential impact on migraine management and healthcare referral (21). This subanalysis was performed to assess whether the sex-based differences observed in the overall sample were consistent across different age groups, and to identify possible age–sex interactions in referral patterns and treatment history.

### Variables included in the study

Clinical and demographic variables such as sex, age at inclusion, time since headache onset, and previously received symptomatic or preventive migraine treatment prior to admission were recorded.

A descriptive and bivariate statistical analysis to evaluate sex differences was carried out using IBM SPSS Statistics version 27.0. For the comparison of categorical variables, the Chi-square test was used, while for continuous variables Mann–Whitney U test was applied. A *p*-value of less than 0.05 was considered statistically significant. The study followed the STROBE (Strengthening the Reporting of Observational Studies in Epidemiology) guidelines (22) for reporting observational research. A complete case analysis was performed, excluding cases with missing values from the corresponding statistical tests.

## Results

**Figure 1:**
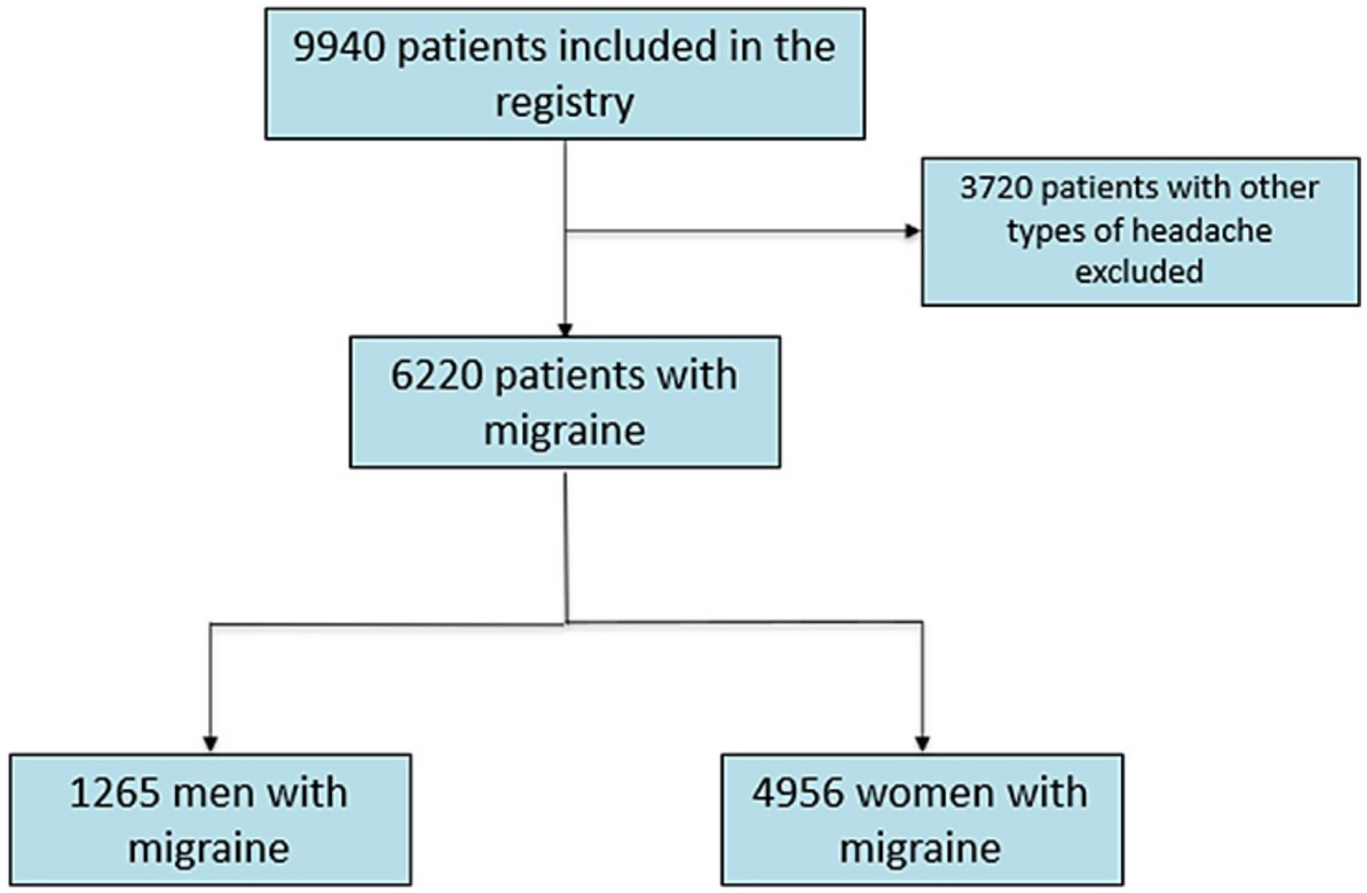
Flowchart of patient selection.

Among 9,940 patients included in the registry, 6,220 had migraine. Regarding sex, 1,264 patients were men (20.3%), and 4,956 were women (79.7%) (Figure 1).

### Demographic characteristics

**Figure 2:**
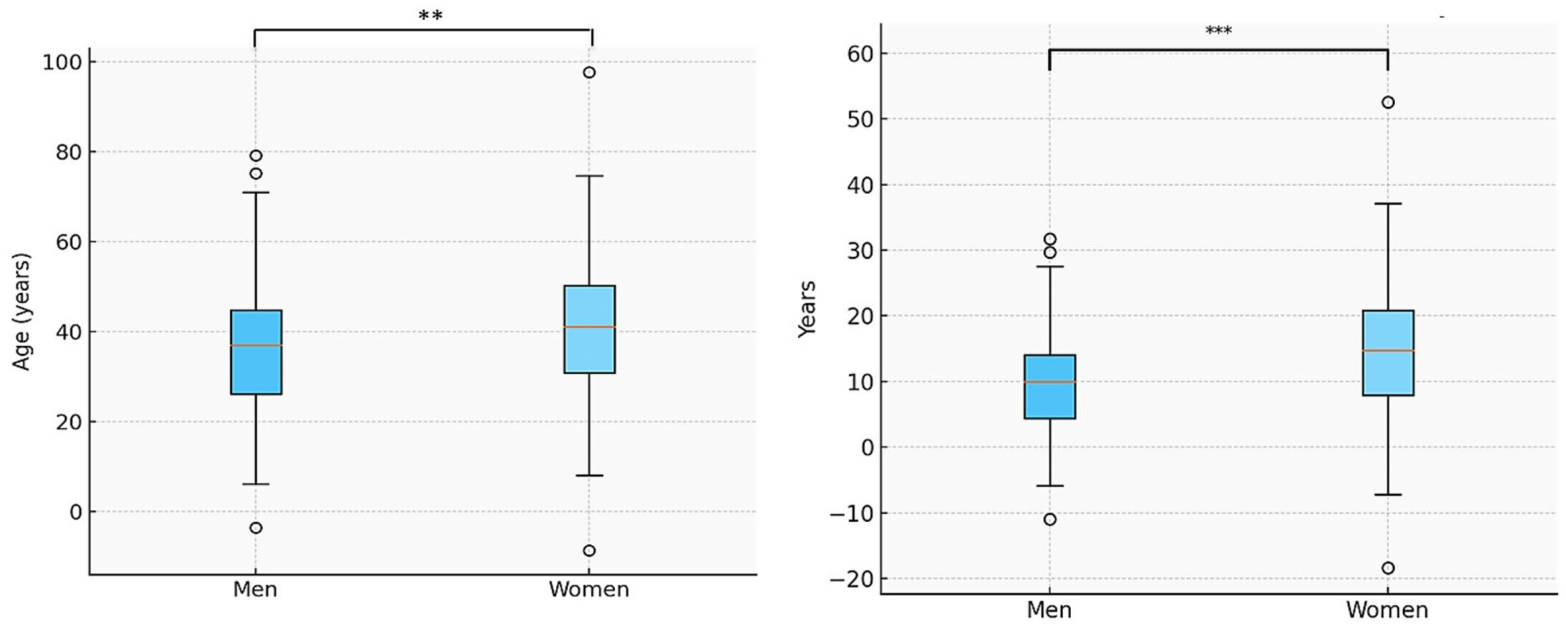
Age at first consultation and years with headache before first visit by sex. Median, interquartile range, and outliers are shown. *p* < 0.01, Mann–Whitney *U* test.

Regarding demographic and clinical variables (Table 1) we observed that age at first visit at the Headache Unit had a higher mean age in female patients compared to male patients (39.93 ± 15.02 vs. 37.07 ± 15.52 years; *p* = 0.004) (Figure 2). However, the age at diagnosis did not differ between groups (22 ± 12.38 vs. 21.76 ± 12.22 years, *p* = 0.486). Demographic and clinical variables are included in Table 1.

**Table 1:** Comparison of demographic and clinical variables between male patients and female patients diagnosed with migraine.

Variable	Male patients	Female patients	*p*-value
Age at first visit (mean ± SD, years)	37.07 ± 15.52	39.93 ± 15.02	0.004
Time from migraine onset to migraine referral (mean ± SD, years)	14.95 ± 14.19	18.11 ± 14.83	< 0.0001
Chronic migraine, *n* (%)	278 (22.0%)	1808 (36.5%)	< 0.0001
Prior use of triptans, *n* (%)	272 (21.5%)	1,421 (28.6%)	< 0.001
Prior preventive treatment, *n* (%)	390 (31.8%)	1891 (39.5%)	< 0.001
Prior antidepressant use, *n* (%)	160 (13.08%)	954 (19.96%)	< 0.001

SD, standard deviation. *p*-values < 0.05 were considered statistically significant. Mann–Whitney U test for continuous variables, Chi-square test for categorical variables.

**Figure 3:**
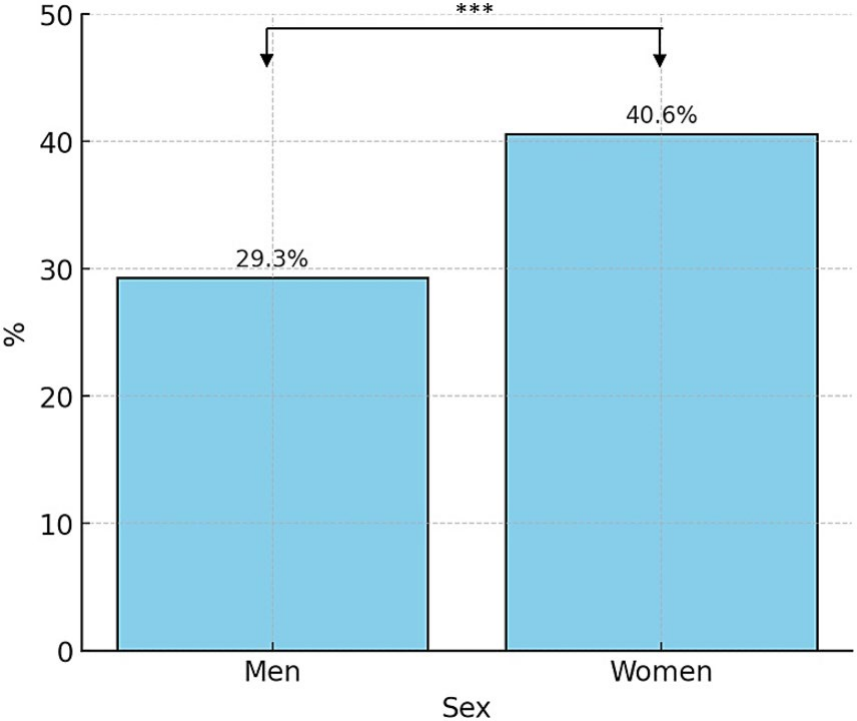
Chronic migraine distribution by sex. Percentage of patients with chronic migraine among male patients (22.0%) and female patients (36.5%). ***p < 0.0001, Chi-square test.

Regarding the time from migraine onset to Headache Unit referral, we observed that it was significantly higher in female patients compared to male patients (of 18.11 ± 14.83 years vs. 14.95 ± 14.19 years; *p* < 0.0001) (Figure 2). Likewise, the percentage of chronic migraine was higher among female patients than male patients, with statistically significant differences (36.5% vs. 22.0%; *p* < 0.0001) (Figure 3).

### Treatment aspects

**Figure 4:**
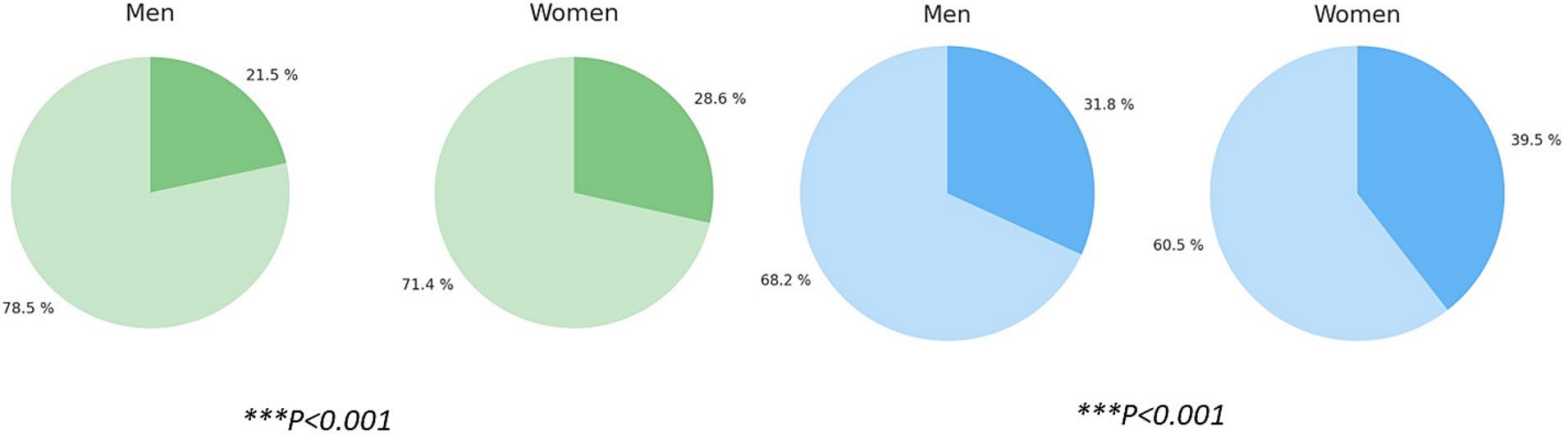
Prior use of triptans by sex, and percentage of patients who received previous preventive treatment by sex. ***p < 0.001, Chi-square test.

Focusing on symptomatic treatment taken before referral to the Unit, we observed that up to 28.6% of female patients had received triptans as symptomatic therapy (Figure 4), while this percentage was substantially lower in the case of male patients (21.5%), making this difference statistically significant (*p* < 0.001).

Considering preventive treatment prior to Headache Unit arrival, 39.5% of female patients reported having received at least one preventive treatment for their migraine while 31.8% of male patients (Figure 4) did, with statistically significant differences (***p < 0.001).

**Figure 5:**
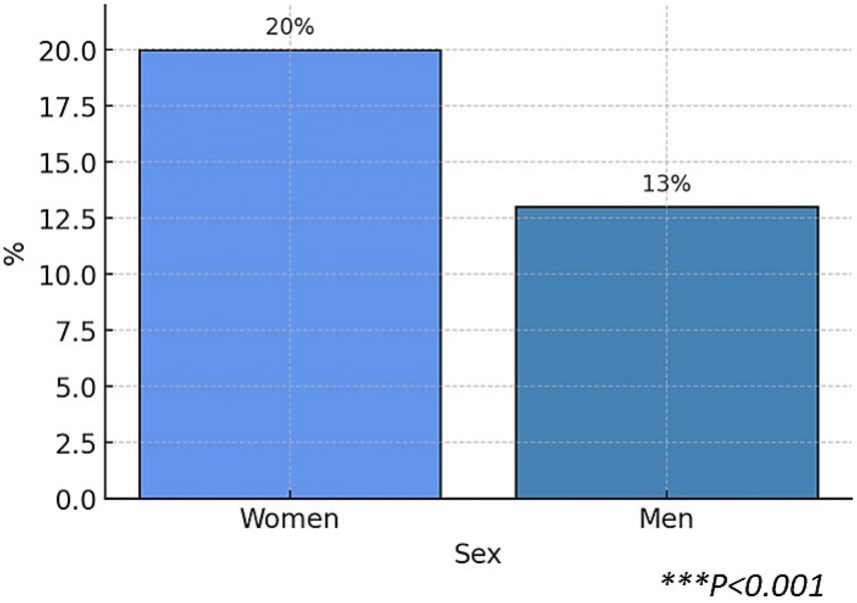
Percentage of patients with prior use of antidepressants as preventive treatment, stratified by sex. Chi-square test, ***p < 0.001.

In addition, regarding preventive treatment, we observed significantly greater prior use of antidepressants among female patients (Figure 5) (954 of 4,779; 19.96%) compared to men (160 of 1,223; 13.08%), with a statistically significant difference (*p* < 0.001).

Multivariable regression analyses were performed to assess the association between sex and referral characteristics as well as prior treatment exposure. Sex was included as the main independent variable. Depending on the outcome, linear regression models were used for continuous variables (age at referral and time since migraine onset), and logistic regression models for binary outcomes (prior triptan use and prior preventive treatment). Models were adjusted for age and chronic migraine. Regression coefficients, odds ratios (ORs), 95% confidence intervals (CIs), and *p*-values were calculated. Full model results are provided in the Supplementary materials. In regression analyses with sex as the independent variable, female patients were referred to the Headache Unit at a later age (≈2–3 years delay) and had longer migraine duration (≈2–3 years more) than male patients, both in crude and adjusted models (*p* < 0.001). They were also more likely to have received triptans (adjusted OR ≈1.3) and preventive treatments (adjusted OR ≈1.2), also in crude and adjusted models (*p* < 0.001). Chronic migraine, prior preventive treatment, and triptan use consistently predicted older referral age, longer disease duration, and higher likelihood of further treatments, whereas referral from primary care was not significantly associated with any outcome.

### Primary care subanalysis

Finally, exclusively considering patients referred by their Primary Care physician, we observed that the aforementioned differences also persist in this subgroup (Table 2). Thus, the age at first consultation (39.32 ± 15.06 vs. 36.02 ± 15.28 years, *p* < 0.001) and time from migraine onset to Headache Unit referral (17.73 ± 14.82 vs. 13.73 ± 13.28 years, *p* < 0.0001) were also higher among female patients, and they also more frequently received triptans (29.4% vs. 21.6%, *p* < 0.001) as well as preventive treatment (34.7% vs. 25.7%, *p* < 0.001) before referral to the Headache Unit. These findings should be interpreted descriptively, as this subgroup analysis does not account for potential temporal changes in referral practices or physician-level variability.

**Table 2:** Comparison of clinical variables between male patients and female patients with migraine referred by primary care physicians.

Variable	Male	Female	*p*-value
Age at first visit (mean ± SD, years)	36.02 ± 15.28	39.32 ± 15.06	< 0.001
Time from migraine onset to migraine referral (mean ± SD, years)	13.73 ± 13.28	17.73 ± 14.82	< 0.0001
Prior use of triptans, *n* (%)	162 (21.6%)	851 (29.4%)	< 0.001
Prior preventive treatment, *n* (%)	192 (25.7%)	1,004 (34.7%)	< 0.001

Statistical significance assessed using Mann–Whitney U test for continuous variables and Pearson’s chi-square test for categorical variables.

### Age-stratified analysis

When stratifying by age groups, no statistically significant differences were observed among patients aged 0–12 years regarding age at first consultation in the specialized headache unit (*p* = 0.375), latency until referral (*p* = 0.813), previous symptomatic treatment with triptans, or prior preventive treatment (*p* = 0.361). It is important to note the small sample size in this subgroup.

In contrast, significant differences were found in the 12–30 age group in terms of age at consultation and referral latency (both *p* < 0.001), as well as in prior use of triptans and preventive therapies (both *p* < 0.001).

Similar findings were observed in the 30–50 age group, with significant differences in age (*p* < 0.001), referral latency (*p* < 0.001), previous triptan use, and prior preventive treatment (both *p* < 0.001).

In the group of patients older than 50 years, statistically significant differences were found in age, referral latency, prior symptomatic and preventive treatment (all *p* < 0.001).

**Figure 6:**
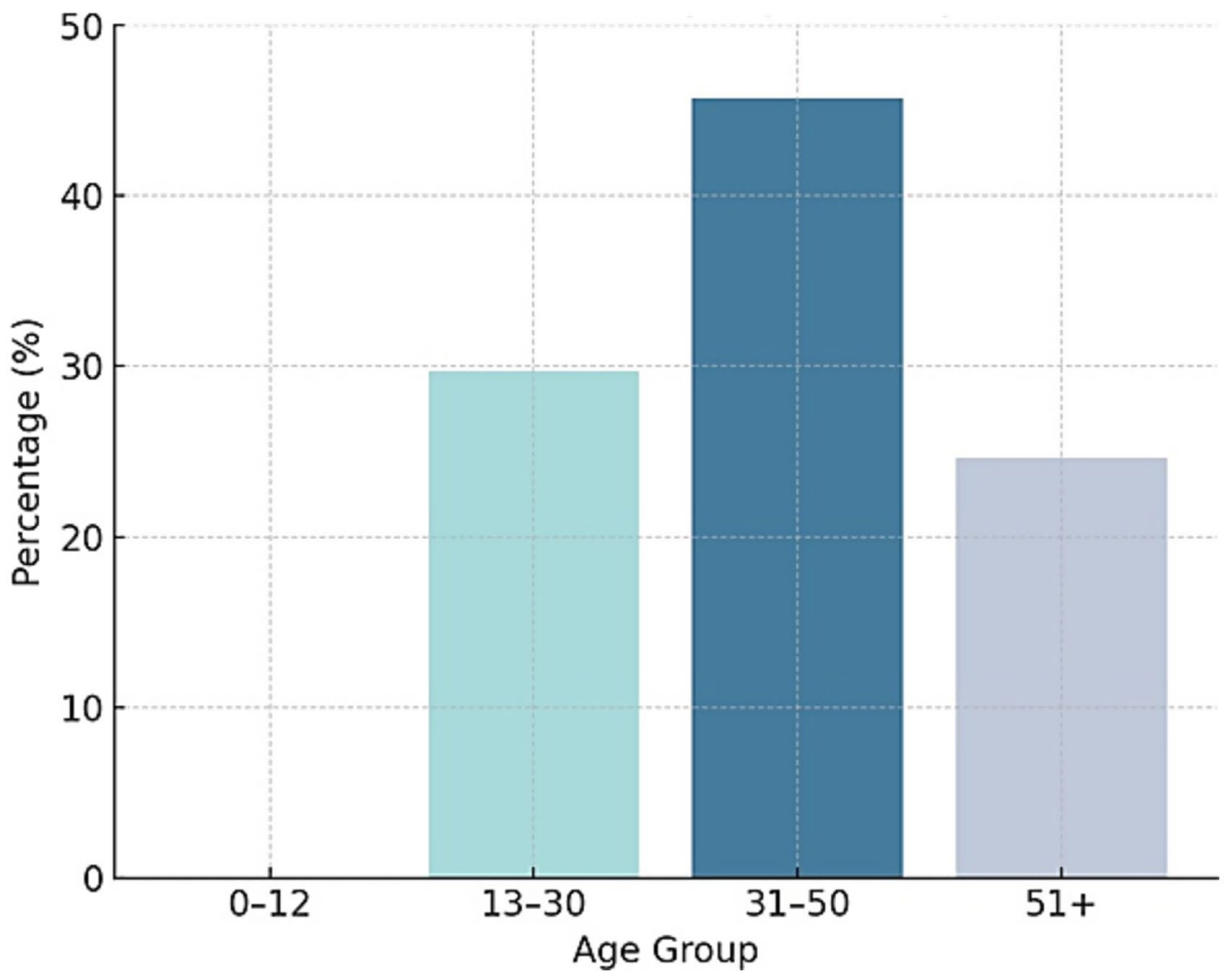
Distribution of patients with migraine referred to the headache unit by age group (years).

As shown in Figure 6, the most represented age groups in the study were 12–30 and 30–50 years, together accounting for 75.4% of the total sample.

### Episodic vs. chronic migraine

Finally, when analyzing the results based on migraine type (episodic, 29% vs. chronic, 71%) significant differences were observed in the episodic migraine group regarding age, referral latency, previous use of triptans, and prior preventive treatment (all *p* < 0.001). In the chronic migraine group, no differences were found in age at referral (*p* = 0.456), but significant differences were observed in referral latency (*p* = 0.036), previous use of triptans (*p* = 0.012), and prior preventive treatment (*p* = 0.01).

## Discussion

Our study found that female were referred to a Headache Unit at an older age and longer disease duration despite having received more frequently symptomatic and preventive treatment. Moreover, there were no differences between patients referred by Primary Care and from the hospital setting. Therefore, our study highlights the sex-differences in migraine treatment pointing out the relevance of sex as an influential modifier in migraine.

Importantly, this study was not designed to measure sociocultural, behavioral, or hormonal variables; therefore, any interpretation regarding these factors should be considered hypothesis-generating rather than confirmatory.

### Sociodemographic characteristics: age and years of headache until referral

We observed that female were, on average, older than men at their first visit to the Headache Unit. This was not explained by later diagnosis. Although migraine onset has been reported as slightly later in female patients (10), this does not account for the older age at referral in our cohort. Puberty begins earlier in girls (23), and the female-to-male prevalence ratio rises sharply after puberty (13), making it paradoxical that women, despite earlier biological development and higher prevalence, are referred later. One explanation may be the higher proportion of chronic migraine among women, which typically develops after years of episodic migraine.

Both the older age and the longer migraine history observed in female patients may also be shaped by sociocultural factors. Women often assume caregiving roles, prioritizing others’ needs over their own (18). Although they generally follow medical advice more closely than men (24), they often delay seeking care for personal health issues (25), usually waiting until symptoms become severe, partly due to time constraints imposed by caregiving and domestic responsibilities.

From the healthcare professional’s perspective, women have traditionally been considered the “weaker” sex (26), supposedly more prone to suffering and complaining. This perception could contribute to medical prejudice (27), leading to less attention being given to women with the same medical problems as men. In contrast, in men, specialized care may be sought earlier either because of atypical clinical presentations that raise greater diagnostic uncertainty (27), or due to the implicit social need of ensuring rapid medical attention for the sex classically regarded as the “more productive” (27), reflecting traditional gender roles, as contemporary society recognizes that nowadays women also participate fully in the productive sphere beyond domestic work.

Hormonal factors may also contribute to sex-related differences in migraine. Estrogen increases trigeminovascular response and susceptibility to cortical spreading depolarization (28), so headaches in female patients are often attributed exclusively to hormonal fluctuations, overshadowing other relevant mechanisms. In addition, fluctuating and declining estrogen levels during perimenopause and menopause are associated with increased pain sensitivity, altered central pain processing, and a higher burden of migraine in a relevant proportion of female patients, potentially contributing to changes in clinical presentation and healthcare-seeking behavior (29). Pregnancy, on the other hand, is associated with higher progesterone levels, which have a neuroprotective effect by reducing trigeminovascular pain transmission (30). With pregnancy occurring at older maternal ages today (31), this may also contribute to later referral of women.

### Treatment management

Regarding treatment, current guidelines make no major distinctions between sexes (18). Nonetheless, both previous studies and our analysis show that female patients more often receive acute and preventive therapies before referral (11). Economically, women account for 80% of the direct costs of migraine, while indirect costs from reduced performance or absenteeism are 50% higher than in men, although these figures vary (18).

The higher proportion of women treated before referral may reflect epidemiology, as migraine is two to three times more common in women (8). Headache in women is therefore more readily linked to migraine, leading to earlier treatment. In men, the lower prevalence may hinder recognition and reduce the likelihood of receiving antimigraine medications. This bias affects both sexes differently: female patients are treated more often but referred later, while men are treated less frequently and possibly with less thoroughness. Advertising and marketing of migraine therapies are also more often directed at women (32), reinforcing gender bias in treatment.

Male patients with migraine are notably less often correctly diagnosed compared with female patients. Whereas typical migraine characteristics tend to be more easily recognized in female patients, atypical or less evident symptoms in male patients often hinder diagnostic accuracy (33). Moreover, gender-related stereotypes may influence this disparity, since men are generally less inclined to verbalize or seek help for their symptoms (34), as doing so may be perceived as conflicting with traditional notions of masculinity. Altogether, these clinical and sociocultural dynamics may contribute to the ongoing underdiagnosis, delayed management, and reduced treatment engagement observed in men.

From a research perspective, male patients remain underrepresented in most clinical trials (18), complicating sex-based comparisons. In our cohort, female patients also had significantly higher prior use of antidepressants. Anxiety and depression are more prevalent in female patients with migraine (35), which may influence both treatment pathways and referral patterns, as well as contribute to stigma.

Ultimately, sex differences in migraine are strongly shaped by sociocultural factors. In general consultations, women are more often treated because migraine is more readily associated with them. However, at the stage of referral to specialized care, they arrive later, at an older age, and with more advanced disease, resulting in greater loss of quality of life.

Our results are consistent with findings from large studies exploring sex differences in migraine. A population-based study reported that migraine and other headache disorders are associated with a higher burden and progression of brain white matter hyperintensities in women compared with men, whereas men with headache showed a comparatively lower neuroimaging burden over time (36). These findings suggest sex-related differences in cumulative disease impact, potentially contributing to a longer and more sustained disease course in women. In parallel, a large national web-based survey addressing sex differences in migraine showed that female patients report higher symptom burden and more frequent use of acute and preventive treatments, whereas male patients are less often treated, report different clinical phenotypes, and display distinct patterns of healthcare utilization (37). Taken together, these observations support our findings that women, despite greater treatment exposure, tend to reach specialized headache care later in the disease trajectory, while men, although referred at a younger age, may remain underdiagnosed and undertreated prior to referral.

This study has several limitations. A major challenge in research on sex differences in migraine is the underrepresentation of male patients in the literature (18), which was also observed in our cohort. Most clinical trials and observational studies predominantly include women, consistent with epidemiology but limiting robust sex-based comparisons.

In addition, this was a single-center study conducted in a tertiary hospital within a specific cultural and healthcare context, which may limit the generalizability of the findings. Larger multicenter studies with broader variable collection are needed to validate these results across diverse populations.

The long inclusion period represents another limitation, as substantial changes in migraine management occurred over time. The introduction of CGRP monoclonal antibodies after 2018 and evolving preventive guidelines may have influenced referral patterns and treatment exposure. As year of referral was not available for adjustment, temporal confounding cannot be fully excluded and may have contributed to some of the observed sex-related differences. Future studies incorporating calendar-period stratification or longitudinal analyses are warranted.

Although missing data cannot be assumed to be completely at random, missingness was less than 20% for all variables used in the primary analyses, which reduces the likelihood of major bias in sex-based comparisons.

The absence of standardized measures of attack frequency, disability, medication overuse, and prior treatment failure limits the ability to fully account for disease severity and healthcare utilization, which may partly explain sex-related differences in referral patterns.

Furthermore, the study was conducted in a referral hospital in central Spain, a region characterized by sociocultural and healthcare contrasts. Rural areas, with older populations and more limited access to specialists, differ substantially from urban settings, where care availability is broader; cultural norms related to gender and illness may also vary. These contextual factors could influence migraine presentation and management, particularly among women.

Finally, although the population was predominantly urban and relatively homogeneous in terms of ethnicity and socioeconomic status, these variables were not included in the analysis, which may have introduced residual bias.

## Conclusion

In our study, female patients with migraine were referred to the Headache Unit at an older age and after a longer disease duration than male patients, despite having received more symptomatic and preventive treatments prior to referral, regardless of referral source. In contrast, male patients were less frequently treated with antimigraine therapies before their first specialized consultation.

These findings reveal sex-related differences in referral and treatment. Female patients are more often treated but reach specialized care later and with more advanced disease, likely reflecting sociocultural norms and gender bias within healthcare systems.

Male patients, although fewer among migraine patients, face a different form of invisibility, being less frequently diagnosed, less treated with targeted therapies, and underrepresented in clinical trials.

Beyond clinical practice, these results underscore the need to improve education and awareness about migraine as a disease affecting both women and men, involving patients, healthcare professionals, the general public, pharmaceutical companies, and the media.

Overall, migraine illustrates how sex-based disparities shape clinical pathways, highlighting the importance of integrating sex as a core dimension of precision medicine to ensure equitable and timely care.

## Data Availability

The original contributions presented in the study are included in the article/Supplementary material, further inquiries can be directed to the corresponding author.
