# Correction: Sex differences in patients' referral to a headache unit

**DOI:** 10.3389/fneur.2026.1793237

**Published:** 2026-04-02

**Authors:** Frontiers Production Office

**Affiliations:** Frontiers Media SA, Lausanne, Switzerland

**Keywords:** sex factors, sex headache management, health care disparities, migraine, referral patterns

In the published article, one of the keywords “gender bias” should be replaced with “sex headache management” The new keywords appear below: sex factors, sex headache management, health care disparities, migraine, referral patterns

A correction has also been made to the **Data availability statement** with the original statement: “The raw data supporting the conclusions of this article will be made available by the authors, without undue reservation” The corrected **Data availability statement** appears below:

**Data availability statement**

“The original contributions presented in the study are included in the article/**supplementary material**, further inquiries can be directed to the corresponding author.”

A correction has also been made to the **Ethics statement** removing the IRB approval: “The studies involving humans were approved by Institutional review board (PI-GR-25-405-APE). The studies were conducted in accordance with the local legislation and institutional requirements. The participants provided their written informed consent to participate in this study”. The corrected Ethics statement appears below:

**Ethics statement**

“Ethical review and approval was not required for the study on human participants in accordance with the local legislation and institutional requirements. Written informed consent for participation was not required from the participants or the participants' legal guardians/next of kin in accordance with the national legislation and institutional requirements.”

A correction has also been made to the **Funding** statement: “GM-A salary has been partially funded by Río Hortega grant Acción Estratégica en Salud from Instituto de Salud Carlos III (CM21/00178) and Juan Rodés fellowship, Subprograma Estatal de Incorporación de la Acción Estratégica en Salud 2023 (JR23/00005)”. The corrected **Funding** statement appears below:

**Funding**

“The author(s) declared that financial support was received for this work and/or its publication. This work was supported by the Instituto de Salud Carlos III (ISCIII), FEDER and FSE funds (grant number CM21/00178, JR23/0005, PI24/01085).”

A correction has also been made to the **Conflict of interest statement**: “The author(s) declared that this work was conducted in the absence of any commercial or financial relationships that could be construed as a potential conflict of interest.” The corrected Conflict of Interest statement appears below:

**Conflict of interest**

“AG-M has received speaker honoraria from TEVA, Abbvie, Lilly and Altermedica. ÁG-P has participated in Advisory Boards: Abbvie, Elly Lilly, Lundbeck, Orga- non, Pfizer, TEVA. Speaker boards: Abbvie, Elly Lilly, Exeltis, Lundbeck, TEVA.

The remaining author(s) declared that this work was conducted in the absence of any commercial or financial relationships that could be construed as a potential conflict of interest.”

A correction is also made to replace “females” to “female patients” and “males” to “male patients”.

The original version of this article has been updated.

