# Supplementary material for: Sex differences in patients’ referral to a headache unit
Source: Front Neurol. 2026 Jan 14;16:1755865. doi: 10.3389/fneur.2025.1755865 (PMC12848913; doi:10.3389/fneur.2025.1755865)
Supplement: Supplementary file 1 [file Table_1.DOCX]

**Supplementary Table S1. Linear regression for age at referral**

| **Variable** | **β (years)** | **95% CI** | **p-value** |
| --- | --- | --- | --- |
| Sex (female vs male) | 2.20 | 1.27 – 3.13 | <0.001 |
| Chronic migraine | 1.84 | 1.38 – 2.29 | <0.001 |
| Prior preventive treatment | 3.27 | 2.46 – 4.08 | <0.001 |
| Prior triptan use | 0.84 | −0.02 – 0.9 | 0.05 |

**Supplementary Table S2. Linear regression for time from migraine onset to referral (years)**

| **Variable** | **β (years)** | **95% CI** | **p-value** |
| --- | --- | --- | --- |
| Sex (female vs male) | 2.06 | 1.18 – 2.94 | <0.001 |
| Chronic migraine | 2.72 | 2.29 – 3.15 | <0.001 |
| Prior preventive treatment | 4.13 | 3.36 – 4.90 | <0.001 |
| Prior triptan use | 3.38 | 2.57 – 4.20 | <0.001 |

**Supplementary Table S3. Logistic regression for prior triptan use**

| **Variable** | **OR** | **95% CI** | **p-value** |
| --- | --- | --- | --- |
| Sex (female vs male) | 1.32 | 1.14 – 1.54 | <0.001 |
| Age at referral (years) | 0.99 | 0.98 – 0.99 | <0.001 |
| Chronic migraine | 1.01 | 0.94 – 1.08 | 0.859 |
| Time since migraine onset (years) | 1.03 | 1.02 – 1.03 | <0.001 |
| Prior preventive treatment | 3.72 | 3.30 – 4.19 | <0.001 |

**Supplementary Table S4. Logistic regression for prior preventive treatment**

| **Variable** | **OR** | **95% CI** | **p-value** |
| --- | --- | --- | --- |
| Sex (female vs male) | 1.12 | 1.1 – 1.29 | 0.05 |
| Age at referral (years) | 1.00 | 0.99 – 1.01 | 0.192 |
| Chronic migraine | 1.50 | 1.40 – 1.60 | <0.001 |
| Prior triptan use | 3.71 | 3.29 – 4.18 | <0.001 |
| Time since migraine onset (years) | 1.02 | 1.01 – 1.02 | <0.001 |
